# Bacteremia as a Cause of Fever in Ambulatory, HIV-Infected Mozambican Adults: Results and Policy Implications from a Prospective Observational Study

**DOI:** 10.1371/journal.pone.0083591

**Published:** 2013-12-30

**Authors:** Troy D. Moon, Wilson P. Silva, Manuel Buene, Luís Morais, Emilio Valverde, Sten H. Vermund, Paula E. Brentlinger

**Affiliations:** 1 Vanderbilt Institute for Global Health, Nashville, Tennessee, United States of America; 2 Friends in Global Health, Maputo, Mozambique; University of North Carolina School of Medicine, United States of America

## Abstract

Fever is typically treated empirically in rural Mozambique. We examined the distribution and antimicrobial susceptibility patterns of bacterial pathogens isolated from blood-culture specimens, and clinical characteristics of ambulatory HIV-infected febrile patients with and without bacteremia. This analysis was nested within a larger prospective observational study to evaluate the performance of new Mozambican guidelines for fever and anemia in HIV-infected adults (clinical trial registration NCT01681914, www.clinicaltrials.gov); the guidelines were designed to be used by non-physician clinicians who attended ambulatory HIV-infected patients in very resource-constrained peripheral health units. In 2012 (April-September), we recruited 258 HIV-infected adults with documented fever or history of recent fever in three sites within Zambézia Province, Mozambique. Although febrile patients were routinely tested for malaria, blood culture capacity was unavailable in Zambézia prior to study initiation. We confirmed bacteremia in 39 (15.1%) of 258 patients. The predominant organisms were non-typhoid *Salmonella*, nearly all resistant to multiple first-line antibiotics (ampicillin, chloramphenicol, and trimethoprim-sulfamethoxazole). Features most associated with bacteremia included higher temperature, lower CD4+ T-lymphocyte count, lower hemoglobin, and headache. Introduction of blood cultures allowed us to: 1) confirm bacteremia in a substantial proportion of patients; 2) tailor specific antimicrobial therapy for confirmed bacteremia based on known susceptibilities; 3) make informed choices of presumptive antibiotics for patients with suspected bacteremia; and 4) construct a preliminary clinical profile to help clinicians determine who would most likely benefit from presumptive bacteremia treatment. Our findings demonstrate that in resource-limited settings, there is urgent need to expand local microbiologic capacity to better identify and treat cases of bacteremia in HIV-infected and other patients, and to support surveillance. Data on the prevalence and susceptibility patterns of important pathogens can guide national formulary and prescribing practices.

## Introduction

Bacterial bloodstream infections cause substantial morbidity and mortality in HIV-infected African patients; case-fatality ratios over 50% have been reported [1]–[8]. The overall prevalence of bloodstream infections in febrile hospitalized African adults has been estimated at 13.5%, with the majority caused by bacteria; the odds ratio for association between HIV infection and bloodstream infection has been reported as 3.4 (p<0.001) in the region based on a systematic review of the available literature [9].

Prior to the introduction of antiretroviral therapy (ART) in Africa, hospital-based studies showed bacteremia to be three times more common and five times more likely to cause death in HIV positive vs. non-HIV positive patients [2], [9]. Marked immunosuppression resulting from delays in seeking or receiving ART likely contributed to this higher bacteremia burden [1], [8], [10].

In most studies, non-typhoid *Salmonella* (NTS) and *Streptococcus pneumoniae* are the predominant infecting organisms [9]. The annual incidence of invasive NTS in HIV-infected African adults may be as high as 7,500/100,000 [11] However, these estimates are based on relatively small numbers of studies, given the size of the continent and the heterogeneity of its HIV-infected subpopulations.

Correct treatment of a bacteremic HIV-infected patient with an effective antimicrobial depends on the clinicianś appropriate decision to treat probable bacteremia presumptively, and their prior knowledge of local patterns of pathogen prevalence and antimicrobial susceptibility. In high income countries, research and microbiologic surveillance have described changes in the epidemiology of bacterial infections in HIV infected patients following the introduction of ART [12]–[15], with greatly decreased occurrence of microbes such as *Pseudomonas*, mycobacteria and NTS [2]. However, microbiology capabilities in sub-Saharan Africa are often limited by infrastructure, cost, and human resource constraints. The absence of microbiologic capacity for diagnosis and/or surveillance outside the main research centers and teaching hospitals forces many clinicians to practice without any locally relevant data about distribution of pathogens, antimicrobial susceptibility patterns, or their evolution in time. Thus, clinicians often must rely on presumptive diagnosis and treatment strategies, such as algorithm-driven clinical decision-making. This can be frustrating, lead to poor clinical outcomes, and promote antimicrobial resistance [9]. For example, it is common practice in much of sub-Saharan Africa to diagnose and treat all fever first as malaria [9], [16]–[20]. However, in one Tanzanian study of hospitalized patients, clinical outcomes were worse for malaria slide-negative patients than slide-positive, suggesting that empiric treatment of malaria alone, without considering other causes of fever such as tuberculosis, bacteremia, and World Health Organization (WHO) Stage III fever, could have contributed to the observed excesses in mortality [19].

Correct identification of the etiology of blood-stream infections may also influence the clinician's management of the febrile patient's HIV infection. Because bacteremia is a WHO stage III HIV/AIDS condition, and recurrent NTS bacteremia is a stage IV/AIDS-defining condition; [21] diagnosis of bacteremia may also influence clinician decisions to initiate ART. Indeed, bacteremic patients who receive both appropriate antimicrobials and ART have improved survival [22].

Thus far, global initiatives for HIV care and treatment scale-up have not prioritized creation of blood-culture or other microbiologic capacity. Rather, lab efforts have focused on HIV diagnosis, CD4+ T-lymphocyte cell count (CD4), complete blood counts (CBC), biochemistries, HIV viral load, and diagnosis of tuberculosis (TB) [23], [24].

Mozambique is a resource-limited country in southern Africa, with a high burden of HIV/AIDS and almost no blood-culture capacity outside a handful of research centers. The prevalence and character of bacterial bloodstream infections has not been described in HIV-infected Mozambican adults; though studies conducted by the Manhiça Health Research Center (*Centro de Investigação em Saúde de Manhiça* [CISM]) have highlighted the importance of bacteremia in hospitalized Mozambican children under 5 years [7], [18], [25]. Thus, Mozambican clinicians have no evidence-based way to estimate the likelihood or etiology of bacteremia in their HIV-infected adult patients, leaving them to guess which parasitic, fungal, and bacterial organisms might be most prevalent locally, and which antibiotics, antimalarials, or other agents might be most suitable for presumptive treatment.

In Mozambique, the scope of the HIV epidemic overlaid with an inadequate number of physicians has resulted in the decentralization of HIV/AIDS care to first- and second-level health facilities, where most care is provided by non-physician clinicians known as *Técnicos de Medicina,* in the context of the international strategy of task-shifting [26], [27]. Mozambique limits the *Técnico de Medicina's* scope of practice in HIV/AIDS care to ambulatory care of patients in WHO HIV/AIDS stages 1 through 3; more complex patients are to be referred to physicians. Most of these non-physician clinicians work in highly resource-constrained peripheral health units. In 2009, the Mozambican Ministry of Health disseminated new guidelines for management of common problems in HIV/AIDS care, including fever, by the *Técnico de Medicina.* The guidelines were intended for use in the management of ambulatory patients at peripheral health units with high caseloads, minimal or absent laboratory and radiologic capacity, and very restricted drug formularies.

**Figure 1:**
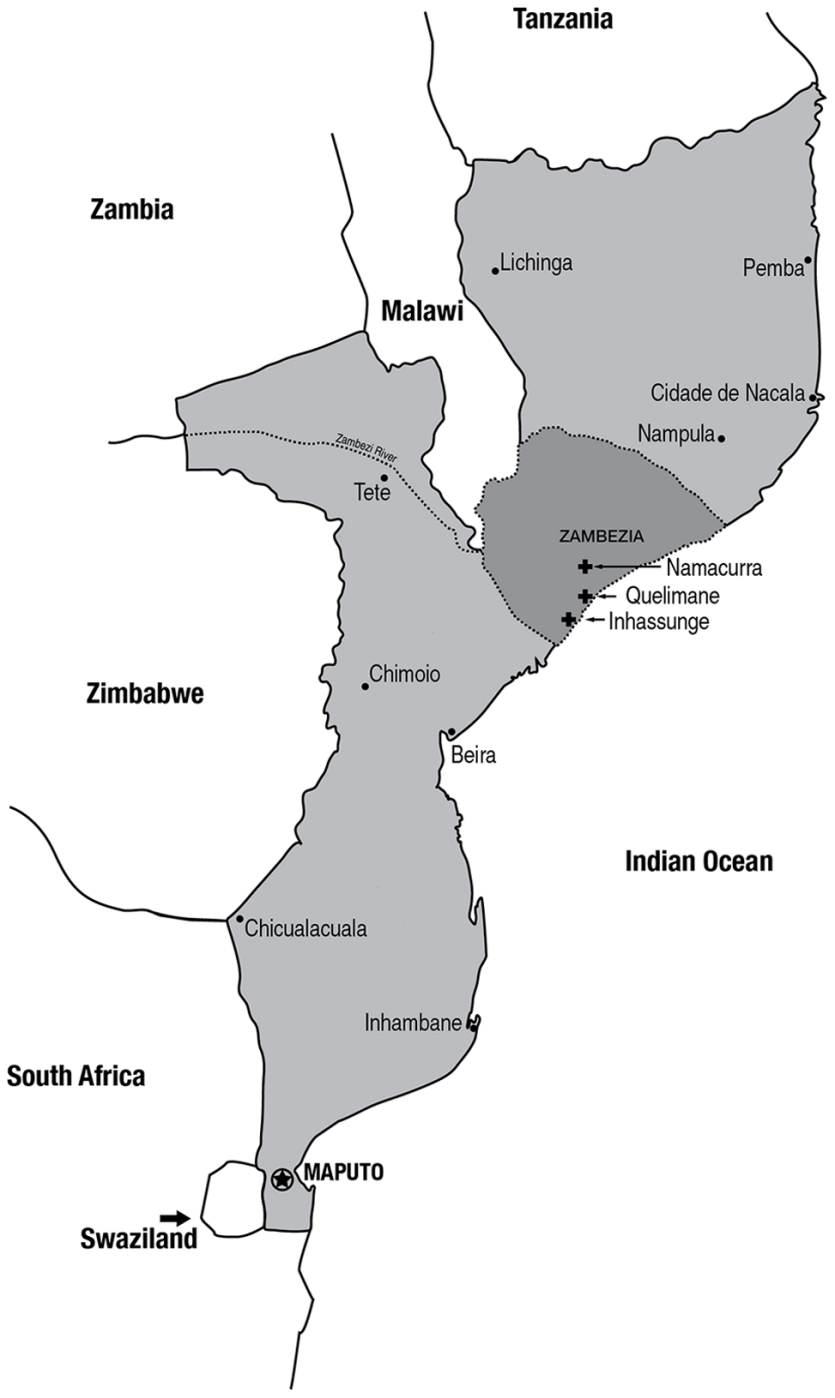
Map of Mozambique with locations of study sites in Namacurra, Inhassunge, and Quelimane City (Coalane Health Center), Zambézia Province.

In this paper, we describe a prospective observational study (clinical trial registration NCT 01681914, www.clinicaltrials.gov) that was conducted between April and September 2012 at 3 health centers, one each in the districts of Inhassunge, Namacurra, and Quelimane in Zambézia Province (Figure 1). All three health centers were resource-constrained rural or peri-urban sites where nearly all ambulatory HIV/AIDS care was provided by *Técnicos de Medicina.* The primary objective of the parent study was to evaluate the performance of new Mozambican guidelines, for non-physician clinician management of fever (or history of fever) and anemia in HIV-infected adults. An important secondary objective, and the focus of this manuscript, was to describe the prevalence, etiology, and clinical correlates of laboratory-confirmed cases of bacteremia.

## Methods

### Context

In 2012, Mozambique ranked 185/187 nations on the Human Development Index of the United Nations Development Programme. Gross national income was estimated at US $906 per capita in 2012 [28], with male and female life expectancies of 47 and 51 years respectively in 2009 [29]. Additionally, Mozambique's total public expenditure on healthcare per capita (2009) was US$50, representing 5.7% of the national gross domestic product, with external resources accounting for 81% of expenditures on health [30]. Mozambique is one of the sub-Saharan African countries most affected by the HIV/AIDS epidemic, with an estimated 2009 national adult (age 15–49) HIV prevalence of 11.5% [31].

Zambézia Province is overwhelmingly rural, located in north-central Mozambique. Its economy is based on subsistence farming and fishing. Zambézia is a designated focus province for both resource allocation and interventions targeting to improve health indices by the Mozambican Ministry of Health (*Ministério de Saúde* [MISAU]), the U.S. Embassy in Mozambique (coordinator of President's Emergency Plan for AIDS Relief [PEPFAR] activities), and the Millennium Challenge Corporation [32]. The countrýs second most populous province, (≈4 million persons), its estimated 2009 adult HIV/AIDS prevalence was 12.6% [31]. The capital city, Quelimane, is the only urban (>50,000 persons) area, with a population of ≈200,000 people and an HIV prevalence that may be two or more times that of the province as a whole, estimated from antenatal sentinel surveillance [33], [34].

### Mozambican HIV/AIDS Treatment Policy

At the time of study enrollment, Mozambican policy approved initiation of co-trimoxazole prophylaxis in HIV-infected adults with CD4 counts <350 cells/μL, active tuberculosis (TB), pregnancy, or WHO clinical stage III or IV. ART was approved for all HIV-infected adults with CD4 counts <350 cells/μL, active tuberculosis (TB), or stage IV opportunistic infections. The standard first-line antiretroviral regimen was zidovudine+lamivudine+nevirapine.

### Mozambique's New Fever Guideline

The 2009 guideline for management of fever or history of fever in HIV-infected ambulatory adults replaced an earlier, very simple guideline that recommended presumptive treatment of malaria for all febrile patients, with hospitalization and the addition of antibiotics for sicker patients. The basic steps in the revised guideline were: 1) Identification of patients with guideline-defined danger signs, who were to be referred to a higher level of care (because of the *Técnico de Medicinás* limited scope of practice); 2) History (including a pre-specified review of systems that included tuberculosis screening questions), physical examination, and malaria testing; 3) Assignment of diagnosis; and 4) Development of a treatment or referral plan. The major differences between the 2009 guideline compared to its predecessor were that malaria testing was obligatory, malaria treatment was strongly discouraged in the absence of a positive malaria test, the pre-specified review of systems and physical exam were more extensive, and the *Técnicos de Medicina* were obliged to consider tuberculosis, other opportunistic infections, and malaria with other concurrent source of fever in their differential diagnosis. The guideline was meant to be applied both to patients with documented temperature elevation (axillary temperature ≥37.5°C) and patients with history of fever in the past 24 hours, because fever associated with malaria (highly endemic in Mozambique) is often intermittent [35].

### Participants and Procedures

**Figure 2:**
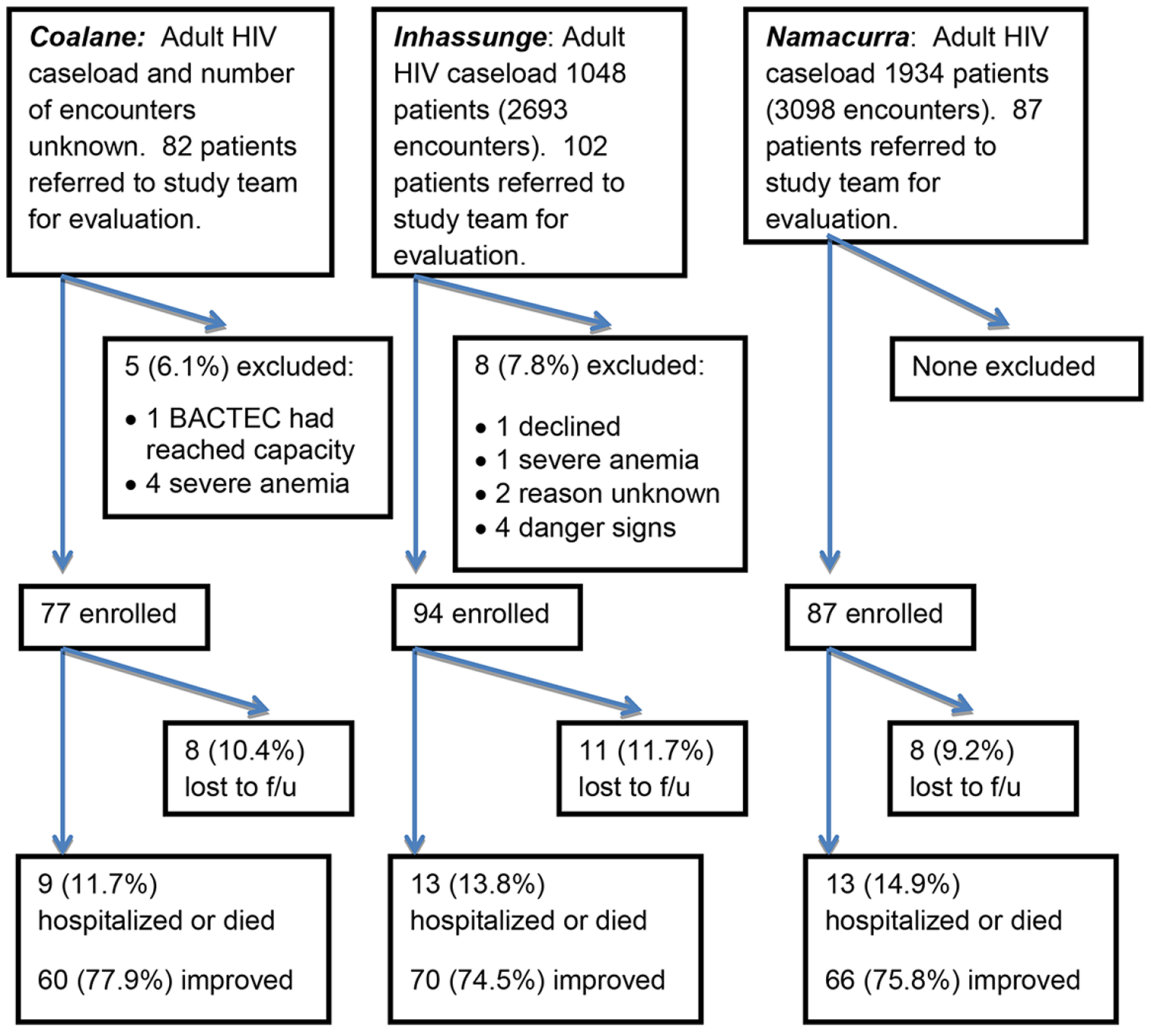
Screening, Eligibility, Enrollment, Outcomes.

HIV-infected ambulatory patients ≥ 18 years of age who presented for routine or urgent care at one of the participating health centers, with an axillary temperature ≥ 37.5°C or history of fever in the preceding 24 hours, were eligible for enrollment (Figure 2). Clinic staff (clinicians, nurses, and counselors) used digital thermometers to measure each patient's temperature and also asked each patient about current or recent fever; they referred patients found to have fever or history of fever to the study team for formal eligibility screening. The screening and enrollment procedures did not prioritize sicker-appearing patients. Subjects were excluded if they presented with guideline-defined clinical “danger signs” (seizures, meningismus, coma, lethargy, other serious changes in behavior or mental status, intense headache, inability to eat, drink or walk, severe abdominal pain, severe dyspnea), at the time of rapid pre-enrollment screening, or if they had previously been treated for the current fever episode. Very ill patients who did not meet the guideline-defined threshold for danger signs but required hospitalization at the first visit were retained in the study for the first visit only. Study staff screened patients Monday to Friday. Random sampling was not logistically feasible. There were no fixed appointments. Most patients arrived in the early morning and their clinical encounters followed at varying intervals, depending on waiting times for vital signs, location of the medical record, adherence counseling, laboratory, and clinical care. Screening of patients for study eligibility was halted daily when the number of patients enrolled corresponded with the available space within the blood-culture instrument (1 or 2 per site daily).

After informed consent and enrollment, blood was collected for a rapid malaria antigen test, hemoglobin, and blood culture. Demographic, HIV staging and treatment history (including co-trimoxazole prophylaxis) were collected on standard study forms. Subjects were referred to study clinicians for determination of the likely cause of fever and appropriate treatment. Study clinicians also addressed other aspects of HIV/AIDS care, e.g. ART. The first scheduled follow-up visit was programmed for 7 days following the initial enrollment visit. Additional sick visits were made as necessary. At each visit, study staff updated the patients medication and laboratory history. Unless the patient opted out of home visits, study field workers did active case finding at the patients' homes when patients failed to return for follow-up visits or blood culture results indicated need for therapy not prescribed at the initial visit. When patients could not be found at home, we also reviewed health-center medical records to determine whether defaulting patients had been seen alive at the health center after the last recorded study visit. Preplanned study endpoints were improvement of fever, hospitalization, death, transfer to a higher level of care, or loss to follow-up. In the context of our parent study, which had the primary aim of assessing the performance of guidelines designed strictly for use in the care of ambulatory patients, hospitalization was deemed a study endpoint because it signaled that the patient required transfer to a higher level of care than that authorized by the *Técnico de Medicina's* scope of practice or that was addressed by the fever guideline.

Although malaria testing is routinely conducted in febrile patients throughout Mozambique, blood culture capacity for bacteremia assessment is extremely limited and was unavailable in Zambézia prior to our study. Because the published literature suggested that bacteremia might be an important contributor to the burden of febrile illness in the study population, we introduced blood-culture capacity in the Quelimane provincial reference laboratory.

### Microbiologic Assessment

At enrollment and upon identification of fever or history of fever, peripheral blood was tested for malaria using the ICT Malaria *Plasmodium falciparum* assay (ICT Diagnostics, Sydney, Australia). Light microscopy examination of thick and thin Giemsa-stained smears was performed for persons with a positive rapid malaria test; each smear was read by 3 microscopists for speciation, calculation of parasite density, and quality control. Hemoglobin was measured using the *HemoCue^TM^ Hb 201+* analyzer (HemoCue Inc. Lake Forest, CA). Quality control of each *HemoCue^TM^* was conducted daily on-site. All symptomatic subjects with positive malaria antigen tests and no history of recent malaria treatment were presumed to have malaria and were treated with antimalarials, because only the first of 3 peripheral smear readings was available at the time of the study visit, because peripheral-smear microscopy may be less sensitive than rapid tests at low levels of parasitemia, and because regional shortages of Giemsa reagents prohibited us from conducting microscopy for 6 patients (15.0%) with malaria antigenemia.

Blood culture specimens were collected on-site by dedicated study personnel laboratory technicians, who were formally trained in collection techniques. To minimize contamination, skin antisepsis was performed using 70% isopropyl alcohol and blood collected through a closed vacutainer system. In brief, blood was collected through a single venipuncture using a butterfly needle with vacuum tubing connected directly to the BACTEC blood culture bottles. Once collected, samples were transported to the Quelimane provincial reference laboratory within 5 hours at room temperature. For each enrolled patient, 8–10 mL of whole blood were inoculated into adult blood-culture bottles (BD BACTEC^TM^ Plus Aerobic/F and Anaerobic/F, Becton-Dickinson, NJ, USA) and incubated in an automated system (BACTEC 9050^TM^ Becton-Dickinson, NJ, USA) for 5 days. If there was no growth at that time, a specimen was identified as a negative culture. Positive samples were examined by Gram stain and sub-cultured on blood, blood chocolate, XLD, or MacConkey agar plates and incubated at 37°C with or without 5% CO_2_, as required. For Gram stain, 50 µL of blood were aliquoted using a sterile 1 mL syringe and needle and spread onto a clean slide to make a smear. After drying and staining, the smear was examined under the microscope. The Gram stain result was used to guide the selection of solid media for subculture of the sample. Gram-positive cocci suggestive of *Staphylococci* and *Streptococci* were subcultured onto 5% sheep blood agar plate and incubated aerobically. Gram positive diplococci suggestive of *S. pneumoniae* were subcultured onto 5% sheep blood agar plate and incubated in 5% CO2. Gram negative bacilli suggestive of *Enterobacteriacea* and related bacteria were subcultured onto 5% sheep blood agar and MacConkey agar plates and incubated aerobically. Gram negative coccobacilli suggestive of *Haemophilus* were cultured onto chocolate agar and 5% sheep blood agar plates and incubated in 5% CO2. Gram negative diplococci suggestive of *Neisseria* were subcultured onto 5% sheep blood agar plates in 5% CO2. All were incubated at a temperature of 37°C.

The plates were analysed for bacterial growth each morning and the characteristics of the colonies were used in combination with the Gram stain result for presumptive identification. *Staphylococci* were identified by the catalase and coagulase tests (Bio-Rad). *Streptococci* were identified by the optochin and bile solubility tests (alpha-haemolytic) or by the *Streptococci* agglutination kit (Bio-Rad), to differentiate between beta- and non-haemolytic *Streptococci* and *Enterococci*. *Enterobacteriacea* and related bacteria were identified using the API 20E strips (bioMerieux). *Salmonella* isolates were typed to determine the serovar using the *Salmonella* agglutination kit (Bio-Rad).

Positive vials were kept at room temperature until the final identification and sensitivity test were performed. We based the determination of blood culture contamination on the identification of the isolated organism, and the number of positive culture bottles [36]. We defined a false positive as a blood culture set that gave a positive Bactec signal but from which no organism could be identified by Gram stain or culture [37].

Antibiotic sensitivity testing was performed using the Kirby-Bauer antibiotic testing method (KB testing or disk diffusion antibiotic sensitivity testing) [38]. The following antibiotics were tested: ampicillin, penicillin, amoxicillin-clavulanate, ceftriaxone, trimethoprim-sulfamethoxazole, gentamicin, nalidixic acid, ciprofloxacin, tetracycline, oxacillin, erythromycin, and chloramphenicol. Isolates were classified as susceptible, intermediate or resistant according to the definitions of the Clinical Laboratory Standards Institute (CLSI) guidelines [39]. For oxacillin-resistant *Streptococcus pneumoniae* strains, penicillin minimum inhibitory concentrations were determined using E-test strips (AB Biodisk^TM^, Solna, Sweden). We did not test for susceptibility to azithromycin.

Once a month, 3 vials inoculated with a 0.5 McFarland suspension of *Staphylococcus aureus (ATCC)* and *Escherichia coli (ATCC)* and a negative vial (inoculated with normal saline) were introduced into the BACTEC 9050^TM^. Quality controls were determined successful if the vials inoculated with the bacteria suspension became positive within 24–72 hours, while the non-inoculated vial remained negative after 5 days. Quality control for antibiotic susceptibility testing was done once a month according to CLSI guidelines [39].

*Salmonella* isolate serotyping (serogrouping and serotyping) was determined at the Quelimane provincial reference laboratory using commercial antisera (Remel, Remel Europe Ltd, UK) following the Kauffmann scheme [40].

### Data Analysis

Clinical and laboratory data were recorded on paper study instruments by the study team and then entered into a password-protected on-line database maintained by the Research Electronic Data Capture (REDCap) Consortium (http://www.project-redcap.org/). The investigators compared each on-line entry to the original paper record to assure the quality of data entry. The data were then converted to Stata 11.2™ (Stata Corporation, College Station, Texas, USA). We described the subjects' baseline characteristics by calculating simple proportions (for dichotomous or categorical variables) or medians with interquartile ranges for continuous variables with non-normal distributions, for all patients and disaggregating by blood-culture result. For initial bivariate analyses, we used logistic regression, with presence of confirmed bacteremia at study visit 1 as the dependent outcome, to compare the characteristics of patients with and without confirmed bacteremia at study visit 1. In these exploratory bivariate analyses, we initially considered both continuous and categorical values of CD4 count, temperature, and hemoglobin. We ultimately elected to retain only categorical variables, because hemoglobin, temperature and CD4 values were not normally distributed, and because (where models were otherwise similar) categorical cutoff values seemed more intuitively useful to working clinicians than the per-unit-change estimates derived from continuous variables. The use of a categorical variable for CD4 also allowed us to create a category denoting unavailable CD4 count, which permitted us to retain 65 subjects with unavailable CD4 results in our models. Covariates associated with bacteremia at a level of p<0.10 were assessed in multivariable logistic regression analyses; we used stepwise backward regression to eliminate covariates that did not retain their statistical significance in multivariable models. In the multivariable model we also included terms for interactions involving CD4 count and antiretroviral status (which proved non-significant), and for interactions involving low hemoglobin and high temperature. The final model retained all covariates significant at a level of p≤0.05. Our own previous analyses (data not shown) had described statistically significant correlations between study site and *P. falciparum* malaria prevalence, but our bivariate models did not confirm the suspected association between malaria and bacteremia, particularly non-typhoid *Salmonella* bacteremia, that has been demonstrated by others [41]–[43]. Therefore, we adjusted for presumed correlation of results within study sites in the final multivariable model rather than incorporating study site as a covariate in the multivariable model.

### Ethical Considerations

Written informed consent was obtained in Portuguese (the national language) or Echuabo (the local language at all three clinics). If the patient was illiterate, informed consent was read in the preferred language and consenting patients marked the informed consent with an “X” in the presence of a witness. Both the Mozambican National Bioethics Committee and the Vanderbilt Institutional Review Board (IRB) approved this protocol.

## Results

We enrolled 258 patients and obtained blood culture results for all. Most patients presented late in the course of HIV infection, and a substantial proportion had not yet initiated ART in spite of clinical and/or laboratory evidence of advanced immunosuppression (Table 1). Of those who had had at least two CD4 measurements and were taking ART at the time of study entry, 17 (6.7% of the 258) had falling CD4 counts. The majority of patients (170 [65.9%]) were on trimethoprim-sulfamethoxazole prophylaxis at the time of enrollment, however. Very recent diagnosis appeared to be common. Of the 236 subjects with available HIV test dates, 73 (30.9%) had been diagnosed within 60 days of study entry.

**Table 1:** Characteristics of Bacteremic and Non-bacteremic Subjects at Fever Visit 1.

		Fever Type at Visit 1		1^st^ Fever Visit:		1^st^ Fever Visit:		1^st^ Fever Visit:	
				All patients		History of Fever		Documented Fever	
Patient		Fever at Enrollment		Bacteremia		Bacteremia		Bacteremia	
Characteristic	All	By History	Documented						
(n [%] or median	patients	T <37.5°C	T ≥37.5°C	No	Yes	No	Yes	No	Yes
[IQR])	(n = 258)	(n = 88)	(n = 170)	(n = 219)	(n = 39)	(n = 81)	(n = 7)	(n = 138)	(n = 32)
*Site:*									
Coalane	77 (29.8)	23 (26.1)	54 (31.8)	63 (28.8)	14 (35.9)	19 (23.5)	4 (57.1)	44 (31.9)	10 (31.3)
Inhassunge	94 (36.4)	37 (42.1)	57 (33.5)	85 (38.8)	9 (23.1)	35 (43.2)	2 (28.6)	50 (36.2)	7 (21.9)
Namacurra	87 (33.7)	28 (31.8)	59 (34.7)	71 (32.4)	16 (41.0)	27 (33.3)	1 (14.3)	44 (31.9)	15 (46.9)
*Sex,pregnancy*									
Fem, Pregnant	11 (4.3)	6 (6.8)	5 (2.9)	10 (4.6)	1 (2.6)	6 (7.4)	0	4 (2.9)	1 (3.1)
Non-pregnant	163 (63.2)	58 (65.9)	105 (61.8)	142 (64.8)	21 (53.9)	54 (66.7)	4 (57.1)	88 (63.8)	17 (53.1)
Male	84 (32.6)	24 (27.3)	60 (35.3)	67 (30.6)	17 (43.6)	21 (25.9)	3 (42.9)	46 (33.3)	14 (43.8)
*Age* (years)	30 (25,38)	32 (25, 38)	30 (26, 38)	30 (25,38)	30 (26, 37)	32 (25, 38)	35 (24, 45)	30 (25, 38)	30 (26, 36)
*CD4 (cells/μL)* *									
<50	26 (10.1)	5 (5.7)	21 (12.4)	17 (7.8)	9 (23.1)	4 (4.9)	1 (14.3)	13 (9.4)	8 (25.0)
50–99	24 (9.3)	6 (6.8)	18 (10.6)	14 (6.4)	10 (25.6)	5 (6.2)	1 (14.3)	9 (6.5)	9 (28.1)
100–199	39 (15.1)	16 (18.2)	23 (13.5)	34 (15.5)	5 (12.8)	15 (18.5)	1 (14.3)	19 (13.8)	4 (12.5)
200–349	37 (14.3)	11 (12.5)	26 (15.3)	35 (16.0)	2 (5.1)	10 (12.4)	1 (14.3)	25 (18.1)	1 (3.1)
350–499	30 (11.6)	17 (19.3)	13 (7.8)	26 (11.9)	4 (10.3)	16 (19.8)	1 (14.3)	10 (7.3)	3 (9.4)
≥500	37 (14.3)	12 (13.6)	25 (14.7)	34 (15.5)	3 (7.7)	12 (14.8)	0	22 (15.9)	3 (9.4)
Missing	65 (25.2)	21 (23.9)	44 (25.9)	59 (26.9)	6 (15.4)	19 (23.5)	2 (28.6)	40 (29.0)	4 (12.5)
*Co-trimoxazole*									
* prophylaxis*	171 (66.3)	69 (78.4)	102 (60.0)	146 (66.2)	25 (64.1)	65 (80.2)	4 (57.1)	81 (58.7)	21 (65.6)
*Antiretroviral*									
* therapy* **	91 (35.3)	31 (35.2)	60 (35.3)	82 (37.4)	9 (23.1)	30 (37.0)	1 (14.3)	52 (37.7)	8 (25.0)
*Eligible, but not*									
* on ART*	106 (41.1)	37 (42.1)	69 (40.6)	84 (38.4)	22 (56.4)	32 (39.5)	5 (71.4)	52 (37.7)	17 (53.1)
*Axillary temp*									
* ≥38.5 °C*	52 (20.2)	N/A	52 (30.6)	36 (16.4)	16 (41.0)	N/A	N/A	36 (26.1)	16 (50.0)
*BMI^$^* (kg/m^2^)	19.5	19.9	19.2	19.6	18.8	19.9	20.2	19.3	18.6
(6 missing)	(17.5,21.3)	(17.9, 21.5)	(17.3, 21.2)	(17.5,21.3)	(17.5, 20.7)	(17.6, 21.5)	(18.0, 21.4)	(17.4,21.3)	(17.3, 20.4)
*Hemoglobin*									
* <8.5 g/dL*									
* (2missing)*	55 (21.5)	20 (23.4)	35 (20.6)	41 (18.9)	14 (35.9)	17 (21.5)	3 (42.9)	24 (17.4)	11 (34.4)
*Active*									
* tuberculosis*	30 (11.6)	11 (12.5)	19 (11.2)	27 (12.3)	3 (7.7)	11 (13.6)	0	16 (11.6)	3 (9.4)
*New diagnosis of*									
* Malaria*	38 (14.7)	14 (15.9)	24 (14.1)	34 (15.5)	4 (10.3)	14 (17.3)	0	20 (14.5)	4 (12.5)
*Oral candidiasis*	14 (5.4)	3 (3.4)	11 (6.5)	8 (3.7)	6 (15.4)	2 (2.3)	1 (14.3)	6 (4.4)	5 (15.6)
*Esophageal*									
* candidiasis*									
* (presumed)*	7 (2.7)	1 (1.1)	6 (3.5)	7 (3.2)	0	1 (1.2)	0	6 (4.4)	0
*Herpes zoster*	7 (2.7)	2 (2.3)	5 (2.9(	7 (3.2)	0	2 (2.5)	0	5 (3.6)	0
*Cough*	178 (69.0)	59 (67.1)	119 (70.0)	148 (67.6)	30 (76.9)	54 (66.7)	5 (71.4)	94 (68.1)	25 (78.1)
*Any upper*									
* respiratory*									
* symptom*	181 (70.2)	66 (75.0)	115 (67.7)	151 (69.0)	30 (76.9)	59 (72.8)	7 (100)	92 (66.7)	23 (71.9)
*Any lower*									
* respiratory*									
* symptom*	172 (66.7)	62 (70.5)	110 (64.7)	146 (66.7)	26 (66.7)	57 (70.4)	5 (71.4)	89 (64.5)	21 (65.6)
*Any*									
* gastrointestinal*									
* symptom*	155 (60.1)	53 (60.2)	102 (60.0)	129 (58.9)	26 (66.7)	48 (59.3)	5 (71.4)	81 (58.7)	21 (65.6)
*Any genitourinary*									
* symptom*	56 (21.7)	19 (21.6)	37 (21.8)	46 (21.0)	10 (25.6)	19 (23.5)	0	27 (19.6)	10 (31.3)
*Headache*	214 (83.0)	71 (80.7)	143 (84.1)	177 (80.8)	37 (94.9)	64 (79.0)	7 (100)	113 (81.9)	30 (93.8)

Measurements represent a CD4 drawn ≤6 months prior to enrollment, or after enrollment if no previous CD4. 128 patients had no reported CD4 count result in the 6 months prior to study enrollment. For patients lacking recent pre-study CD4 results, we used the first CD4 drawn after enrollment when available. 65 patients had no CD4 result from either time period.

Excludes patients on 1 or 2-drug regimens for prevention of mother to child transmission (PMTCT).

$BMI =  Body Mass Index.

Of the 258 patients included in the analyses, 39 (15.1%) had confirmed bacteremia. The predominant organisms (Table 2) were NTS, nearly all of which were resistant to multiple first-line antibiotics (ampicillin, chloramphenicol, and trimethoprim-sulfamethoxazole). One patient with a culture positive for coagulase negative *Staphylococcus* was classified as true bacteremia rather than contamination, based on clinical characteristics and course [44]. Two additional patients had positive growth in the BACTEC, but subsequently showed no growth on culture and were not counted as bacteremic (one patient subsequently was diagnosed with smear positive pulmonary tuberculosis and the other was a pregnant woman with slide-confirmed malaria). One blood culture was classified as contaminated and not included in the bacteremia analyses.

**Table 2:** Identification and Antimicrobial Susceptibility Patterns of 39 Blood Culture Isolates.

Organism	N (%) Isolates Sensitive											
	Nalidixic Acid	Ampicillin	Amoxicillin- Clavulanate	Ceftriaxone	Ciprofloxacin	Chloramphenicol	Co- trimoxazole	Erythromycin	Gentamicin	Oxacillin	Penicillin	Tetracycline
*E. coli* (n = 2)	1 (50)	0 (0)	2 (100)	2 (100)	2 (100)	1(50)	0 (0)		2 (100)			2 (50)
*Salmonella*												
NOS (n = 1)	1 (100)	1 (100)	1 (100)	1 (100)	1 (100)	1 (100)	0 (0)		1 (100)			0 (0)
*Salmonella*												
*enteriditis*												
(n = 1)	1 (100)	1 (100)	1 (100)	1 (100)	1 (100)	1 (100)	1 (100)		1 (100)			1 (100)
*Salmonella*,												
Group B												
(n = 2)	1(50)	0 (0)	0 (0)	2 (100)	2 (100)	0 (0)	0 (0)		2 (100)			2 (100)
*Salmonella*												
*typhimurium*												
(n = 27)	26(96.3)	1(3.7)	16(59.3)	27(100)	23(85.2)	1(3.7)	1(3.7)		25(92.6)			27(100)
Coagulase												
Negative												
*Staphlococcus*												
(n = 1)		0 (0)	1 (100)								0 (0)	
*Strep*												
*pneumoniae*												
(n = 4)		2 (50)				3 (75)	0 (0)	3 (75)		2(50)	2(50)	2(50)
*Strep*												
*pyogenes,*												
group A												
(n = 1)		1(100)		1(100)		1(100)		1(100)			1(100)	1(100)

Ten (25.6%) of 39 bacteremic patients were hospitalized, five at 1^st^ study visit and five after the positive blood culture prompted home visits (Table 3). The five admitted after active follow-up had all deteriorated clinically since the initial study visit, either because the antibiotic prescribed was a poor match for the antimicrobial susceptibility pattern or because an appropriate antibiotic had not been dispensed owing to pharmacy stock-outs. One patient with *E. coli* bacteremia defervesced on appropriate antibiotics but then died with progressively worsening anemia. We documented 27 (69.2%) bacteremic persons to have improved clinically. One failed to return for the first follow-up visit but was confirmed to have survived. By comparison, only 24 (11.0%) of the non-bacteremic patients died or were hospitalized (p = 0.01 for comparison of these adverse outcomes in bacteremic vs. non-bacteremic patients by χ^2^ test). During active follow-up, we were able to ascertain the post-study vital status of all but six of the non-bacteremic patients who failed to return for planned study visits. Had all six died or been hospitalized, adverse outcomes would still have been twice as common in bacteremic patients compared to non-bacteremic patients.

**Table 3:** Patient Outcomes.

Outcome (n, %)	All subjects	No bacteremia	Bacteremic
	(n = 258)	(n = 219)	(n = 39)
Hospitalized or died	35 (13.6)	24 (11.0)	11 (28.2)
Improved	196 (76.0)	169 (77.2)	27 (69.2)
Lost to follow-up	27 (10.5)	26 (11.9)	1 (2.6)

Both bacteremic and non-bacteremic patients had multiple other comorbidities, including anemia, tuberculosis, malaria, herpes zoster, fungal infections, and untreated HIV disease (Table 1). Of those with malaria and available peripheral smear results, 97% had both malaria antigenemia and confirmed *P. falciparum* parasitemia on microscopy. In both bivariate and multivariate models of associations between different clinical and laboratory characteristics and confirmed bacteremia (Table 4), the clinical characteristics most significantly associated with bacteremia included lower CD4 count, higher temperature, lower hemoglobin, and headache. (Overall, six patients with headache were hospitalized because they also had mental status changes and/or meningeal signs; two of them were bacteremic). The combination of high temperature and low hemoglobin was also significantly associated with bacteremia. Untreated HIV patients (ART eligible but not yet on ART) and oral candidiasis were associated with bacteremia in bivariate models but were not retained in the multivariate model after adjustment for CD4 count. Bivariate models also suggested associations of bacteremia and both study site and ART status (Table 4). Although higher temperature was associated with bacteremia, lower temperature did not rule bacteremia out since 7 of the bacteremic patients (18.0%) had axillary temperatures ≤37.3°C. The presence of malaria antigenemia was not statistically associated with bacteremia, although all four patients with concurrent malaria and bacteremia had NTS bacteremia (three of the four had confirmed *P. falciparum* parasitemia).

**Table 4:** Correlates of Bacteremia at Fever Visit 1. Bivariate and Multivariable Logistic Regression.

Patient Characteristic	Bivariate (n = 258 except as noted)		Multivariable (n = 256)*****	
	OR (95% CI)	P value	OR (95% CI)	P value
Site				
Coalane	2.10 (0.85, 5.15)	0.106		
Inhassunge (Reference)	–	–		
Namacurra	2.13 (0.89, 5.11)	0.091		
Gender/Pregnancy categories				
Female, pregnant	0.39 (0.05, 3.29)	0.390		
Female, non-pregnant	0.58 (0.29, 1.18)	0.132		
Male (Reference)	–	–		
Age (years)	1.00 (0.96, 1.03)	0.909		
Current CD4<100 cells/μL (vs. higher or no	5.76 (2.77,12.00)	<0.001	5.52 (1.23, 24.88)	0.026
CD4)				
Co-trimoxazole prophylaxis	0.91 (0.45, 1.86)	0.798		
Antiretroviral therapy (excludes PMTCT**)	0.50 (0.27, 1.11)	0.088		
Untreated AIDS***	2.08 (1.04, 4.14)	0.037		
Axillary temperature ≥38.5 °C	3.54 (1.70, 7.35)	0.001	3.59 (0.61, 21.14)	0.157
Hemoglobin <8.5 g/dL (2 missing)	2.40 (1.15, 5.02)	0.020	3.91 (1.69, 9.04)	0.001
T≥38.5 °C AND Hemoglobin <8.5 g/dL****			7.45 (1.21, 45.88)	0.030
Body mass index (kg/m^2^) (6 missing)	0.93 (0.82, 1.04)	0.198		
New diagnosis of malaria	0.62 (0.21, 1.86)	0.396		
Oral candidiasis	4.80 (1.56, 14.70)	0.006		
Cough	1.60 (0.72, 3.55)	0.248		
Any upper respiratory symptom	1.50 (0.68, 3.33)	0.318		
Any lower respiratory symptom	1.00 (0.49, 2.06)	1.000		
Any gastrointestinal symptom	1.40 (0.68, 2.86)	0.363		
Any genitourinary symptom	1.30 (0.60, 2.85)	0.518		
Headache	4.39 (1.02, 18.94)	0.047	5.67 (1.26, 25.47)	0.023

Measurements represent a CD4 drawn ≤6 months prior to enrollment, or after enrollment if no previous CD4.

PMTCT =  Prevention of mother to child transmission.

Eligible for ART but not on ART (based on CD4 or TB status).

The odds ratio and 95% CI for the interaction term (low hemoglobin + high temperature) were 0.53 (0.07, 4.20).

The multivariable model is adjusted for presumed correlation of results within 3 study sites.

Prescribing practices of the study clinicians changed early in the course of the study, after the first bacteremia cases were reported. Once the first dozen cases of bacteremia were confirmed, we reviewed the clinical records of bacteremic patients and identified lower hemoglobin, higher temperature, lower body mass index (BMI), and lower CD4 count as apparent risk factors. We encouraged clinicians to treat presumptively for bacteremia (with ciprofloxacin or azithromycin) in patients with this profile, but unfortunately the small patient numbers available early on did not permit us to establish specific cutoff points (for hemoglobin, temperature, CD4, or BMI) that would suggest treatment to be definitively indicated. All clinicians were aware of our interim findings by July 1, 2012. Prior to that date, 30.3% of our fever patients were treated presumptively for bacteremia at the first fever visit, vs. 62.3% afterwards (p<0.001 by χ^2^ test).

## Discussion

Before we began our study, local clinicians had never seen confirmed cases of bacteremia, because there was no blood-culture capacity. Introduction of blood cultures specifically to support our study allowed us to accomplish the following: 1) We confirmed the presence of bacteremia, an unfamiliar diagnosis, in a substantial proportion of our febrile patients; 2) We tailored specific antimicrobial therapy based on results of antibiotic susceptibility testing; 3) We made informed choices of presumptive antibiotics for patients with suspected bacteremia based on analysis of antibiotic susceptibility patterns; and 4) We constructed preliminary clinical profiles of bacteremic patients to help clinicians determine which patients would be most likely to benefit from presumptive antimicrobial treatment effective against common local pathogens.

Perhaps because of our policy of active case-finding for patients with positive blood cultures and use of early antimicrobial susceptibility patterns to inform clinicians about antibiotic selection, we had encouraging clinical results. Only one patient death was reported during the bacteremia episode, and 33 (86.3%) of 39 bacteremic subjects were confirmed to have recovered from the index episode of bacteremia, although we do not know their long-term (e.g., 6-month) outcomes. We believe undetected bacteremia may be an important cause of preventable mortality in Mozambican HIV/AIDS patients, particularly those with advanced disease who have not yet accessed ART; this is consistent with observations from Malawi and Kenya [22], [45].

The 15.1% observed prevalence of bacteremia in our ambulatory, adult HIV-infected subjects was unexpectedly high. It exceeded the 8.1% prevalence of bacterial blood stream infections found in a cohort of symptomatic Malawian adults presenting for ART, and was similar to the prevalence reported in some hospital-based studies involving patients of unknown or mixed HIV serostatus [9], [46]. Had our study included all patients presenting with guideline-specified clinical danger signs, our point estimates for bacteremia prevalence would presumably have been even higher. However, we were obliged to exclude such patients from participation in the current study because the primary aim of our parent study was to describe the performance of the Mozambican fever guideline when used by *Técnicos de Medicina* to care for febrile HIV-infected adult patients, and MISAU policy does not permit non-physician clinicians to manage patients with danger signs, or hospitalized patients.

The inclusion of patients without objective fever did not necessarily limit our findings. The fever guideline specifies that it is to be applied to patients with documented temperature elevation or history of fever in the past 24 hours because malaria, an important cause of febrile illness in Mozambique, often presents with intermittent fever [35]. Exclusion of patients with history of fever (but no documented temperature elevation) might have biased the results towards an underestimation of malaria cases, and would also have prohibited us from comparing the prevalence of bacteremia in patients with history of fever vs. patients with documented temperature elevation. Indeed, exclusion of patients without documented temperature elevation would have excluded 18% of our bacteremic patients and 34% of our subjects overall.

Because ours was not a longitudinal cohort, we were unable to estimate incidence rates and so are not able to compare our results to bacteremia incidence reported in Uganda [2]. The rainy season lasted longer than usual in Zambézia in 2012, and because *Salmonella* transmission has been observed to be seasonal, it is possible that our observed prevalence overestimated the seasonal average in this population.

The distributions of bacterial pathogens and antibiotic susceptibility patterns observed in our study cannot be presumed to be generalizable, even within Mozambique, though NTS has been among the predominant causes of bacteremia in multiple previous studies of bacterial bloodstream infections in HIV-infected Africans [9], [11], [22], [47]. NTS has also been a significant problem in HIV-infected patients in Southeast Asia and in the United States [48]–[52]. To the best of our knowledge there have been no other published descriptions of bacteremia in HIV-infected Mozambican adults. We expect both the prevalence and antimicrobial susceptibility profiles of both NTS and other agents may vary somewhat both geographically and temporally within Mozambique.

We observed a high prevalence of multi-drug resistance in both NTS and other isolates. This is consistent with many other regional reports of multi-drug resistant bacterial pathogens, including NTS in Malawi and southern Mozambique, *S. typhi* in Tanzania, and urinary-tract pathogens in Beira, Mozambique [53]–[56]. This is especially alarming as it suggests that the relatively high coverage of trimethoprim-sulfamethoxazole prophylaxis in our subjects (65.9% at enrollment) is not protective against locally common, potentially life-threatening pathogens. In our sample, 29/31 (93.5%) of NTS isolates were resistant to trimethoprim-sulfamethoxazole, as were 4/4 (100%) of *S. pneumoniae* isolates.

Late initiation of ART, which has been previously described in Mozambique [33], [57], almost certainly contributed to the high observed burden of bacteremia. In our bivariate analyses (Table 4), both untreated AIDS and lower CD4 count were significantly and positively associated with bacteremia. In bivariate analyses, only the variable for lower CD4 count was retained, even after adjustment for ART status. Although we were unable to document time on ART systematically owing to lacunae in medical records, we believe – based on review of cases with known ART start times – that this phenomenon can be explained by the substantial number of patients who had initiated ART very recently and had not yet had a chance to achieve immune reconstitution, and the smaller but still substantial number who had falling CD4 counts on ART but did not have access to second-line regimens. The combination of late (or no) initiation of ART and inadequate local capacity to diagnose and treat stage III and IV opportunistic infections appears ominous for this patient population.

An unanticipated adverse consequence of the discovery of multiple cases of bacteremia in the earliest phase of our study was a marked increase in presumptive antibiotic prescribing by the study clinicians. Although our patients responded well to ciprofloxacin, azithromycin, and ceftriaxone, significant levels of NTS resistance to these agents has been documented in Asia, and is likely to occur in southern Africa as well if antibiotics are not used wisely [58], [59]. An additional concern is the possibility that presumptive use of ciprofloxacin might result in the development of drug-resistant tuberculosis if inadvertently given to a patient whose true diagnosis was TB [60], [61]. In the absence of a sensitive and specific rapid test for bloodstream infections, Mozambican clinicians have no current alternative to presumptive antibiotic prescription in bacteremia suspects. There is an urgent need to develop evidence-based antibiotic prescribing guidelines that would maximize the likelihood that bacteremic patients would receive effective presumptive treatment, while minimizing overuse of antibiotics. The observed associations between bacteremia and higher temperature, lower hemoglobin, and lower CD4 count in our study subjects may be of use in the development of clinical prediction rules for bacteremia diagnosis. However, future development of such clinical prediction rules should be based on aggregation of data from larger and more nationally representative samples. Our sample was not strictly random, and reported p-values and confidence intervals for association between patients' clinical characteristics and bacteremia status must therefore be interpreted cautiously.

We believe that our findings demonstrate that in Mozambique, as elsewhere, there is a pressing need to expand local microbiologic capacity – especially bacterial and mycobacterial blood-culture capacity – so that clinicians may better identify and treat individual cases of bacteremia (and AIDS with bacteremia as the presenting diagnosis) in HIV-infected patients. Establishment of blood and other culture capacity would also help Mozambique to develop its capacity for ongoing surveillance, which is not feasible at present owing to the nationwide paucity of culture capacity. Longitudinal data on the prevalence and antimicrobial resistance patterns of important pathogens are badly needed to guide national policy for drug formulary expansion and antibiotic-prescription guidelines. In April, 2012, Mozambique's Minister of Health authorized the creation of a Technical Working Group for the Revitalization of Microbiology Laboratories in Mozambique. This working group has recently declared that its core aims include creation of culture and sensitivity capacity – including blood cultures – at the country's provincial hospitals [62]. We believe that this proposed expansion in MISAU's microbiology capacity would result in a substantial reduction in the burden of preventable mortality in both HIV-infected and uninfected Mozambicans.
